# A three-arm single blind randomised control trial of naïve medical students performing a shoulder joint clinical examination

**DOI:** 10.1186/s12909-021-02822-5

**Published:** 2021-07-21

**Authors:** P. E. Brewer, M. Racy, M. Hampton, F. Mushtaq, J. E. Tomlinson, F. M. Ali

**Affiliations:** 1 Sheffield Teaching Hospitals, Northern General Hospital, Herries Road, Sheffield, S57AU UK; 2 University of Sheffield, Western Bank, Sheffield, S10 2TN UK; 3 University of Leeds, Leeds, Ls2 9JT UK; 4 Chesterfield Royal Hospital, Derbyshire, Chesterfield, S44 5BL UK

**Keywords:** Distance learning, Physical examination, Technology, Teaching, Medical education

## Abstract

**Background:**

Technological advances have previously been hailed as a new dawn in Higher Education, with the advent of ‘massive open online courses’ (MOOCs) and online learning. Virtual platforms have potential advantages such as accessibility and availability but simply transferring educational material to the online environment may not ensure high quality learning. Clinical examination is a fundamental principle of medical assessment, and this study aimed to assess the role of technology in teaching these skills.

**Aims/objectives:**

To determine whether three teaching modalities were of equal efficacy in teaching examination of the shoulder joint to naïve medical students.

**Methods:**

Sixty-seven pre-clinical medical students naïve to large joint examination were recruited. Participants completed a learning style questionnaire and were then block randomised to three study: textbook study, face-to-face seminar, or video tutorial via online platform. The same examination technique was taught in all groups, with the intervention being the method of delivery All second year students were eligible for inclusion. The single exclusion criteria was previous exposure to clinical examination teaching. Students were assessed using a standardised scoring system at baseline (pre-intervention), and days 5 and 19 post-intervention (maximum score 30). Assessors were blinded to group allocation. The primary outcome was assessment score at day 5 post intervention.

**Results:**

There was no difference between the three groups at baseline assessment (mean scores 2.4 for textbook, 2.8 for face-to-face, and 3.1 for video; *p* = 0.267). Mean post-intervention scores were 16.5 textbook, 25.5 face-to-face, and 22.4 video (*p* < 0.001, η^2^ = .449). There was no change between day 5 and day 19 post-intervention assessment scores in any group (*p* = 0.373), Preferred learning style did not affect scores (*p* = 0.543).

**Conclusion:**

Face-to-face teaching was the most effective method for teaching clinical examination of the shoulder. Technology can potentially increase accessibility and remove geographic barriers, but is not as effective if teaching techniques are simply mirrored in an online format.

Online platforms allow in depth data analysis of how learners interact with educational material and this may have value in improving the design of online educational materials, and is a potential area for further research.

**Supplementary Information:**

The online version contains supplementary material available at 10.1186/s12909-021-02822-5.

## Introduction

Taking a detailed history and performing a thorough clinical examination have always been the foundation to making a diagnosis. Good clinical examination skills are thought to increase the quality of care and reduce cost [1, 2]. Investigations are performed as an adjunct to confirm or refute the differential diagnoses. Undergraduate medical education has sought to instill these skills at the earliest opportunity. The core principles of teaching a clinical examination have not evolved significantly over the last 100 years and is typically taught via a combination of large group didactic and small group seminar- based teaching styles [3]. Simulated encounters have aided as an adjunct to support this [4].

Several studies have shown that clinical examination skills are often lacking in both undergraduate and postgraduate trainees [1, 5–10]. There is also a lack of agreement over the ideal time to teach clinical examination skills, and more importantly the most effective method. Particular concerns have been raised over the quality of musculoskeletal clinical skills teaching [7, 11–14]. The discrepancy between teaching time and proportion of patients seen with musckuloskeletal conditions is striking; Oswald et al. cite in Canada 2.25% of time in the curriculum is devoted to teaching the musculoskeletal system examination skills versus up-to 20% of presentations in primary care relating to the musculoskeletal system [7]. Furthermore, Freedman et al. found only 18% of post graduates had exposure to musculoskeletal medicine and 86% of those surveyed had low confidence performing these examination skills. This shows a potential problem in both the quantity of time allocated to teaching in an undergraduate setting, and the exposure to reinforce this a a post graduate [15].

Modern technology means educational content can be hosted online and accessed via multiple devices. They can be accessed at times that suit the learner and support new forms of pedagogy e.g. reverse classrooms [16]. Education via technological platforms is however not without difficulty, with only 10% of those enrolling for ‘Mass Online Open Courses’ (MOOCs) completing the course. This however may not be generalisable to the well-motivated and goal-orientated medical student. There are a multitude of reasons for this, with many of them yet to be successfully addressed [17]. It is clear that simply recording taught content and hosting it online is not enough to fully engage learners, and that social adaptation of technology does not immediately translate to education [18].

Our primary study outcome is to assess the effectiveness of different teaching modalities on naïve medical students performing a clinical examination of the shoulder joint. This randomised control trial is the first to directly compare seminar small group teaching, textbook learners and a custom-made video tutorial via an online platform.

## Methods

### Design

This study was a prospective randomised trial comparing three different teaching modalities to teach clinical examination skills to second year medical students. The study was designed to compare three different methods of teaching clinical examination skills - face to face teaching, a custom-made educational video and a textbook chapter. An identical examination technique was taught to each group, with the mode of delivery being chosen as the intervention. Detailed information on the intervention in each group is given below.

The content was standardised across each modality. After permission from Cambridge University Press the Shoulder examination chapter from Examination Techniques in Orthopaedics [19] was reproduced in three formats.

Once participants had been recruited there were asked to complete a ‘VARK’ learning styles questionnaire and then block randomised by a computer random number generator into the three intervention groups.

The intervention in each group was on day zero of the study, to ensure standardisation of timelines. Participants underwent a formal assessment of their clinical examination skills before randomisation, and at day 5 and day 19 post intervention. Participants were asked to examine a patient without shoulder pathology, and were assessed by an examiner blinded to the intervention type, using a standardised scoring matrix.

The study was undertaken in the medical school building then students were in a familiar environment, and was conducted in the evenings to avoid timetable clashes with scheduled teaching. The study ran for 21 days from baseline assessment to final assessment.

### Recruitment

Through opportunity sampling, we aimed to recruit second year medical students at the University of Sheffield (cohort size *n* = 290) to the study. The only exclusion criterion was previous teaching on musculoskeletal examination. The study proposal was presented to the entire year group, and contact details provided for the research team. Students were emailed by the University following the presentation and provided with the contact details for the research team. The students were provided with an information booklet detailing the aims and objectives of the study and outlined the requirements. Students wishing to enroll in the study were asked to complete a consent form for the study. University of Sheffield ethical approval was obtained for the study (Ref Number 013097). All methods were performed in accordance with the approved proposal. All were screened for previous musculoskeletal examination teaching, and none of those recruited had any previous teaching, meaning no students were excluded.

All candidates completed a ‘VARK’ questionnaire [20] ascertaining their dominant learning style and all performed a baseline assessment. The VARK questionnaire measures four perceptual preferences of learning style, visual, auditory, reading/writing and kinesthetic. There are 16 questions exploring preferences and the total score indicates the individuals preferred learning style. This method has been shown to be valid and reliable [21]. The baseline assessment examined each candidate performing a shoulder examination on a patient with no shoulder pathology. They were assessed against a pre-determined marking scheme. The assessment tool comprised of six domains marked out of 30; inspection (6 marks), palpation (3 marks), movement (5 marks), rotator cuff (4 marks), special tests (6 marks), joint above/below and neurovascular assessment (3 marks), correct sequence (3 marks).

Based on the candidates learning styles, they were block-randomised by a computer random number generator into three intervention groups- textbook, seminar session and access to a custom-made online video. All three versions followed a consistent sequence and exhibited the same techniques.

### Textbook group

This group were given a copy of the Shoulder examination chapter from the ‘Examination Techniques in Orthopaedics’ textbook. The chapter contains both written and pictorial descriptions of a standardised approach to examination of the shoulder, and describes the technique developed and taught by one of the senior authors. Study participants in this group all received their own copy of the chapter and were free to annotate this as required for their own learning.

### Video group

A custom-made video showing the senior author teaching the same comprehensive shoulder examination was developed. The video was recorded in studio conditions by a cameraman with significant experience in making surgical educational video resources. This was then uploaded to an online platform (SproutVideo) which allowed each candidate to have an individual password protected log-on. The platform allows advance analytics - who accessed the video, what time of day it was accessed, what device it was watched on (i.e. smart phone versus laptop), and which parts of the video were watched.

The video was in exact replica of the technique covered in the textbook chapter, with the examination sequence matched to that described in the textbook chapter.

### Face to face seminar

The third group attended a face-to-face teaching session lasting 30 min, delivered by the senior author teaching 10/ 11 candidates per session. This session was held at the University of Sheffield Medical School to ensure learners were in a familiar learning environment. The examination sequence followed the exact method used in both the textbook chapter and the custom video. The senior author demonstrated the clinical examination technique and tests on a healthy volunteer. Students did not practice or receive feedback. The teaching sequence and method was an exact replication of the educational video used in the study.

Candidates were explicitly told not to access other groups teaching modality (i.e. share log-on for access to the online video) but could practice and use other resources available to try and mirror real life practice for a students’ assessment.

Five participants withdrew from the study. As this was a voluntary study no further action was taken and the study continued as per protocol. No outcome data was collected for these participants.

### Statistical analysis

To ensure that the three groups showed equivalent ability prior to the intervention, we performed a one-way ANOVA (Video vs. Textbook vs. Seminar) on baseline scores. To examine the impact of each intervention, we calculated the difference in performance change relative to baseline for the Assessment on Day 5 and on the Retention Test on Day 19. This performance change measure served as the dependent variable for a 2 (Phase: Assessment vs Retention) × 3 (Training Group: Seminar vs. Textbook vs. Video) mixed ANOVA. We used simple effects to decompose interactions and report Bonferroni corrected *p* values for post-hoc comparisons. To explore the contribution of learning style, we recapitulated the analysis using a mixed effects model with Intervention Group and Stage (Assessment vs Retention) as fixed effects and subject and sex as random effects. We then compared this model to one that included VARK score as a fixed effect to see if the inclusion of learning styles would provide greater explanatory power of the results. We set an alpha threshold of .05 for statistical significance, but given the notable limitations of this approach [22–24], we report eta squared (η^2^) and Cohen’s *d* for effect sizes and include measures of standard error and 95% confidence intervals so that readers are able to more accurately interpret the extent of any differences. Statistical analyses were performed using R version 3.0.2, with the mixed model analysis implemented using the “lme4” package [25].

## Results

**Fig. 1:**
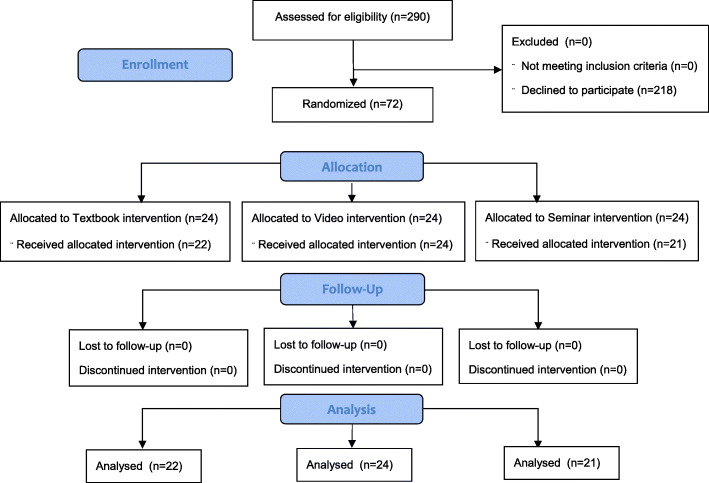
Consort trial flow diagram showing recruitment and retention into the trial

A total of 72 students were recruited to participate in the study, with no exclusions. Five students did not attend for the baseline assessment and dropped out. The mean age of the 67 candidates who completed the study was 21 years (SD = 2.03) (range 19 to 27) and 37 participants (55%) identified as female. Figure 1 shows the participant flow through the trial.

A total of 67 participants completed the study. There were 24 participants in the video group, 22 in the textbook group and 21 in the face-to-face teaching group.

Inter-rater reliability of the performance scores, examined through having six of the candidates independently assessed by two examiners at the same time, indicated strong agreement between the assessors (κ = .839, *p* < .001).

A comparison of pre-test examination scores showed no differences between the training groups (F [2,64] = 1.37, *p* = .262, η^2^ = .041; Seminar Group Mean = 2.76; SE = .248; 95% CI 2.28–3.25; Textbook Group Mean = 2.36; SE = .345; 95% CI 1.69–3.04; Video Group Mean = 3.13; SE = .368; 95% CI 2.4–3.85). The 2 (Phase: Assessment vs Retention) X 3 (Training Group: Seminar vs. Textbook vs. Video) showed no effect of Phase (F [1,64] = .804, *p* = 0.373, η^2^ = .002) and there was no Phase X Group interaction (F [2,64] = .16, *p* = .852, η^2^ = .005). However, we did find a main effect of Group (F [2,64] = 26.1, *p* < 0.001, η^2^ = .449).

**Fig. 2:**
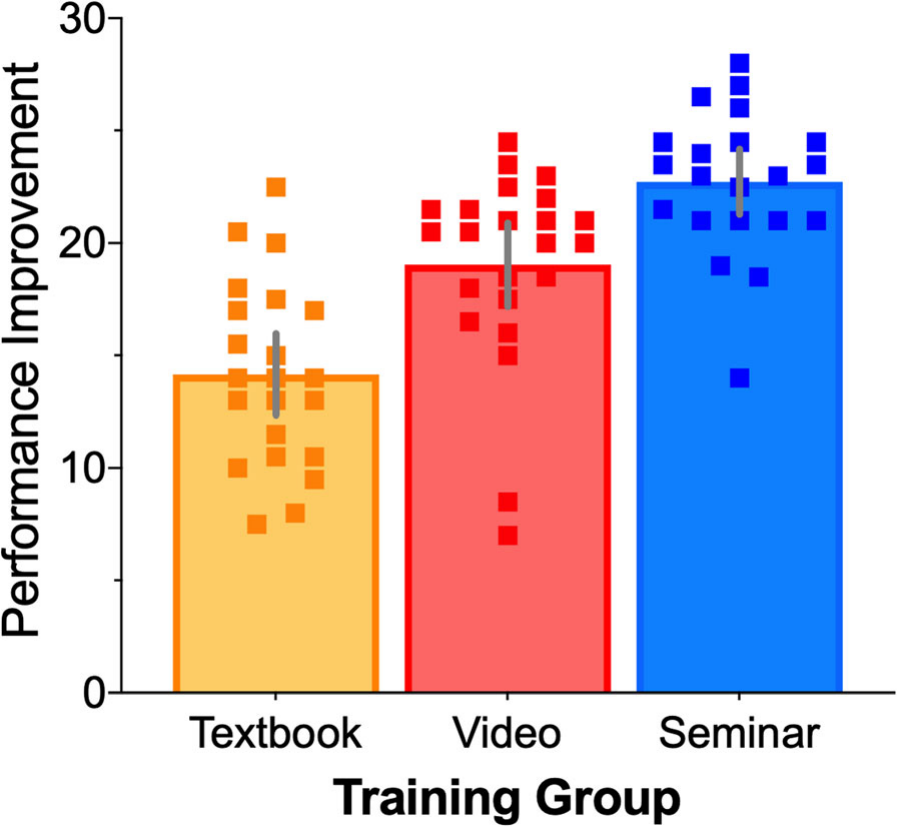
Average performance improvement across Training groups. The error bars represent 95% confidence intervals and each square indicates an individual participant’s average performance across Assessment and Retention

Post-hoc Bonferroni corrected comparisons revealed statistically reliable differences across all three comparisons. The Seminar Group (Estimated Marginal Mean = 22.7; SE = .839; 95% CI 21.0–24.4) scored higher (t = 2.98, *p* = .012, *d* = 0.75) than the Video Group (Estimated Marginal Mean = 19.2; SE = .82; 95% CI 17.6–20.8) and the Textbook Group (Estimated Marginal Mean = 14.1; SE = .832; 95% CI 12.5–15.8; t = 7.17, *p* < .001, *d* = 1.79). In turn, the Video Group significantly (t = 4.4, *p* < .001, *d* = 1.1) outperformed the textbook group. To provide an intuitive visualization of the difference across groups, we collapsed scores across Assessment and Retention and plot the average performance improvement between Groups in Fig. 2.

Finally, there was no statistically reliable impact on the results based on the participants learning styles, as a mixed model with learning styles included as a fixed effect showed no superiority over one without (X^2^ = 9.07, *p* = .247).

## Discussion

This study demonstrated there was no influence of learning styles on learning, but there was an effect of intervention type, with face-to-face teaching being the most effective way to teach clinical examination of the shoulder. Notably, there was no difference in performance between initial post intervention assessment and retention test 2 weeks later.

Multiple studies have shown that physical examination skills are lacking in both undergraduate and postgraduate settings. ^12 = 14^ Given the importance of these skills in delivering high quality care and reducing the cost of care [2] it is critical to understand the ideal way to teach these skills. Previous work by Kurihara et al. suggested no difference between interventions in their four arm randomised trial [26]. This study suggests that face-to-face teaching remains the gold standard and is the most effective way to teach shoulder examination to naive medical students when compared to a custom-made educational video.

Educational videos have the advantage of being accessible at any time, from any location from either a mobile phone, tablet, or computer. Despite this accessibility it was less effective than a single face-to-face teaching session. This study particularly highlights the difference between ‘learning’ and ‘teaching’ [27] as each group were taught in a different way and yet how this was learned or practiced in small groups or individually prior to the testing may have varied. Despite balanced groups prior to the intervention the learning style played no role in the outcome and the method was significant. We hypothesize this to be related to the engagement and interaction with the learner with reinforcement throughout their contact time. Lave and Wenger [28] has previously described the importance of dialogue and social interaction in learning and this is likely to play a role in the superior outcomes seen with face-to-face teaching.

Of the cohort included in this study 100% had a smart phone and therefore had a wealth of information at their fingertips to learn from for their undergraduate education. The advantages offered by such resources include the fluid and ever-evolving content; textbooks can be outdated by the time they are published and distributed. Additionally, they can be viewed during opportune moments without the need to pre-plan. An example of how unreliable online resources can be has recently been published by Singh et al. [29]; they found of over 100 “YouTube” educational videos on rheumatoid arthritis only 55% were accurate and 30% were misleading. This inaccuracy emphasizes the importance of directing the learner to valuable and reliable resources.

A great deal of controversy surrounds the VARK questionnaire [30]. Fleming et al. [31] linked learning styles with four main categories; visual, auditory, reading/ writing and kinesthetic. This method has been validated [21] but evidence shows that changing the way in which material is taught to align learning style does not improve outcomes enough to justify the financial outlay [32, 33].

The balance of power has shifted over the last 10 years; the medical student is now a consumer of medical education and a customer of the Medical School. Massive increases in the cost of attending medical schools in the UK means the demand for good quality teaching is as high as it has ever been, hence the need for good quality teaching which is proven to be effective.

Cost analysis was not specifically examined during this study, but we estimate our video would cost £5000 for professional filming and production. If this was to be expanded to all systems examinations or ‘simple procedures’ the cost may impede their production. However, looking to the future, for examination techniques one would not expect this to evolve significantly over time and therefore after the initial effort and expense of making a bespoke video it then could be reused for the foreseeable future.

To our knowledge this study is the only randomised control trial comparing a custom made, internet hosted educational video to traditional methods for teaching orthopaedic clinical examination skills. The major strength of this study is the robust methodology and adherence to protocol for each candidate once randomised. The blind assessment and high consistency between our independent assessors add confidence to the reliability of the results.

The limitations of this study are the inherent bias associated with the enthusiasm and motivation of those students voluntarily enrolling in a ‘clinical examination’ study. The importance of an instructor who is enthusiastic and knowledgeable also plays a significant role in information retention and cannot be underestimated.

Given the widespread adoption of virtual platforms for meetings and education, exacerbated the pandemic, there is a need for further research comparing live teaching in face to face or via an online platform such as Zoom. Looking further into the future, there are a plethora of emerging technologies such as virtual and augmented reality that are showing a great deal of promise for delivering undergraduate healthcare education [34–40]. The need for alternative solutions to face-to-face delivery has been exacerbated by the COVID-19 pandemic [41] and we suggest that the successful implementation of these technologies will rely on how well they are able to mimic the key features of face-to-face teaching. The present work shows that this approach remains the gold standard for teaching joint examinations.

## Conclusion

Face-to-face teaching is the most effective way to teach clinical examination of the shoulder to previously naive medical students, with significantly superior scores on blinded assessments when compared with a custom-made educational video or reading a textbook. These results indicate that clinical examination skills should be taught with face-to-face teaching where possible.

## Supplementary Information

**Additional file 1.**

## Data Availability

The data sets used and analysed during this study are available from the corresponding author on reasonable request.
