# Supplementary material for: A three-arm single blind randomised control trial of naïve medical students performing a shoulder joint clinical examination
Source: BMC Med Educ. 2021 Jul 21;21:390. doi: 10.1186/s12909-021-02822-5 (PMC8293563; doi:10.1186/s12909-021-02822-5)
Supplement: Supplementary file 1 — Additional file 1. [file 12909_2021_2822_MOESM1_ESM.pdf]

VARK
visual aural read/write kinesthetic

The VARK Questionnaire (Version 7.8)

How Do I Learn Best?

Choose the answer which best explains your preference and circle the letter(s) next to it.
Please circle more than one if a single answer does not match your perception.
Leave blank any question that does not apply.

1. You are helping someone who wants to go to your airport, the center of town or railway station. You would:
   - a. go with her.
   - b. tell her the directions.
   - c. write down the directions.
   - d. draw, or show her a map, or give her a map.
2. A website has a video showing how to make a special graph. There is a person speaking, some lists and words describing what to do and some diagrams. You would learn most from:
   - a. seeing the diagrams.
   - b. listening.
   - c. reading the words.
   - d. watching the actions.
3. You are planning a vacation for a group. You want some feedback from them about the plan. You would:
   - a. describe some of the highlights they will experience.
   - b. use a map to show them the places.
   - c. give them a copy of the printed itinerary.
   - d. phone, text or email them.
4. You are going to cook something as a special treat. You would:
   - a. cook something you know without the need for instructions.
   - b. ask friends for suggestions.
   - c. look on the Internet or in some cookbooks for ideas from the pictures.
   - d. use a good recipe.
5. A group of tourists want to learn about the parks or wildlife reserves in your area. You would:
   - a. talk about, or arrange a talk for them about parks or wildlife reserves.
   - b. show them maps and internet pictures.
   - c. take them to a park or wildlife reserve and walk with them.
   - d. give them a book or pamphlets about the parks or wildlife reserves.
6. You are about to purchase a digital camera or mobile phone. Other than price, what would most influence your decision?
   - a. Trying or testing it.
   - b. Reading the details or checking its features online.
   - c. It is a modern design and looks good.
   - d. The salesperson telling me about its features.
7. Remember a time when you learned how to do something new. Avoid choosing a physical skill, eg. riding a bike. You learned best by:
   - a. watching a demonstration.
   - b. listening to somebody explaining it and asking questions.
   - c. diagrams, maps, and charts - visual clues.

d. written instructions – e.g. a manual or book.

8. You have a problem with your heart. You would prefer that the doctor:
   a. gave you a something to read to explain what was wrong.
   b. used a plastic model to show what was wrong.
   c. described what was wrong.
   d. showed you a diagram of what was wrong.

9. You want to learn a new program, skill or game on a computer. You would:
   a. read the written instructions that came with the program.
   b. talk with people who know about the program.
   c. use the controls or keyboard.
   d. follow the diagrams in the book that came with it.

10. I like websites that have:
    a. things I can click on, shift or try.
    b. interesting design and visual features.
    c. interesting written descriptions, lists and explanations.
    d. audio channels where I can hear music, radio programs or interviews.

11. Other than price, what would most influence your decision to buy a new non-fiction book?
    a. The way it looks is appealing.
    b. Quickly reading parts of it.
    c. A friend talks about it and recommends it.
    d. It has real-life stories, experiences and examples.

12. You are using a book, CD or website to learn how to take photos with your new digital camera. You would like to have:
    a. a chance to ask questions and talk about the camera and its features.
    b. clear written instructions with lists and bullet points about what to do.
    c. diagrams showing the camera and what each part does.
    d. many examples of good and poor photos and how to improve them.

13. Do you prefer a teacher or a presenter who uses:
    a. demonstrations, models or practical sessions.
    b. question and answer, talk, group discussion, or guest speakers.
    c. handouts, books, or readings.
    d. diagrams, charts or graphs.

14. You have finished a competition or test and would like some feedback. You would like to have feedback:
    a. using examples from what you have done.
    b. using a written description of your results.
    c. from somebody who talks it through with you.
    d. using graphs showing what you had achieved.

15. You are going to choose food at a restaurant or cafe. You would:
    a. choose something that you have had there before.
    b. listen to the waiter or ask friends to recommend choices.
    c. choose from the descriptions in the menu.
    d. look at what others are eating or look at pictures of each dish.

16. You have to make an important speech at a conference or special occasion. You would:
    a. make diagrams or get graphs to help explain things.
    b. write a few key words and practice saying your speech over and over.
    c. write out your speech and learn from reading it over several times.
    d. gather many examples and stories to make the talk real and practical.

The VARK Questionnaire Scoring Chart

Use the following scoring chart to find the VARK category that each of your answers corresponds to. Circle the letters that correspond to your answers

e.g. If you answered b and c for question 3, circle V and R in the question 3 row.

Question	a category	b category	c category	d category
3	K	V	R	A

Scoring Chart

Question	**a** category	**b** category	**c** category	**d** category
1	K	A	R	V
2	V	A	R	K
3	K	V	R	A
4	K	A	V	R
5	A	V	K	R
6	K	R	V	A
7	K	A	V	R
8	R	K	A	V
9	R	A	K	V
10	K	V	R	A
11	V	R	A	K
12	A	R	V	K
13	K	A	R	V
14	K	R	A	V
15	K	A	R	V
16	V	A	R	K

Calculating your scores

Count the number of each of the VARK letters you have circled to get your score for each VARK category.

Total number of **V**s circled =	
Total number of **A**s circled =	
Total number of **R**s circled =	
Total number of **K**s circled =	
